# Using LiST to model potential reduction in under-five mortality in Burkina Faso

**DOI:** 10.1186/1471-2458-13-S3-S26

**Published:** 2013-09-17

**Authors:** Andrew Marsh, Melinda Munos, Banza Baya, Djeneba Sanon, Kate Gilroy, Jennifer Bryce

**Affiliations:** 1 Department of International Health, Johns Hopkins Bloomberg School of Public Health, 615 N. Wolfe St., Baltimore, MD, USA; 2 Institut National de la Statistique et de la Démographie, Avenue Pascal Zagré, Ouagadougou, Burkina Faso; 3 Ministry of Health, Ougadougou, Burkina Faso; 4 IntraHealth, Washington, DC, USA

## Abstract

**Background:**

Under-five mortality remains high in Burkina Faso with significant reductions required to meet Millennium Development Goal 4. The Acceleration for Maternal, Newborn, and Child Health is being implemented to reduce child mortality in the North and Center North regions of Burkina Faso.

**Methods:**

The Lives Saved Tool was used to determine the percent reduction in child mortality that can be achieved given baseline levels of coverage for interventions targeted by the Acceleration. Data were obtained from the Demographic and Health Survey 2003, the Multiple Indicator Cluster Survey 2006, and the baseline survey for the program from 2010. In addition to the scale up, scenarios were generated to examine the outcome if secular trends in intervention coverage change persisted and if intervention coverage levels remained constant.

**Results:**

Scaling up all interventions to their target coverage level showed a potential reduction in under-five mortality of 22 percent, with district specific reductions in mortality ranging from 14 to 25 percent. The percent reduction in under-five mortality that might be attributable to the program was 16 percent and varied between 14 and 19 percent by district. Treatment of diarrhea with ORS and malaria with ACTs accounted for the majority of the reduction in mortality.

**Conclusions:**

These findings suggest that significant reductions in under-five mortality may be achieved through the scale-up of the Acceleration. The Ministry of Health and its partners in Burkina Faso should continue their efforts to scale up these proven interventions to achieve and even exceed target levels for coverage.

## Background

Burkina Faso remains a country with high under-five mortality. According to the Demographic and Health Survey (DHS) of 2003 the under-five mortality rate was 184 deaths per 1,000 live births, with mortality in rural areas reaching 202 deaths per 1,000 live births [1]. Results from the 2010 DHS confirm that under-five mortality has been declining [2], but significant reductions in mortality must be achieved for Burkina Faso to meet its Millennium Development Goal 4 target of 68 deaths per 1,000 live births [3].

**Figure 1:**
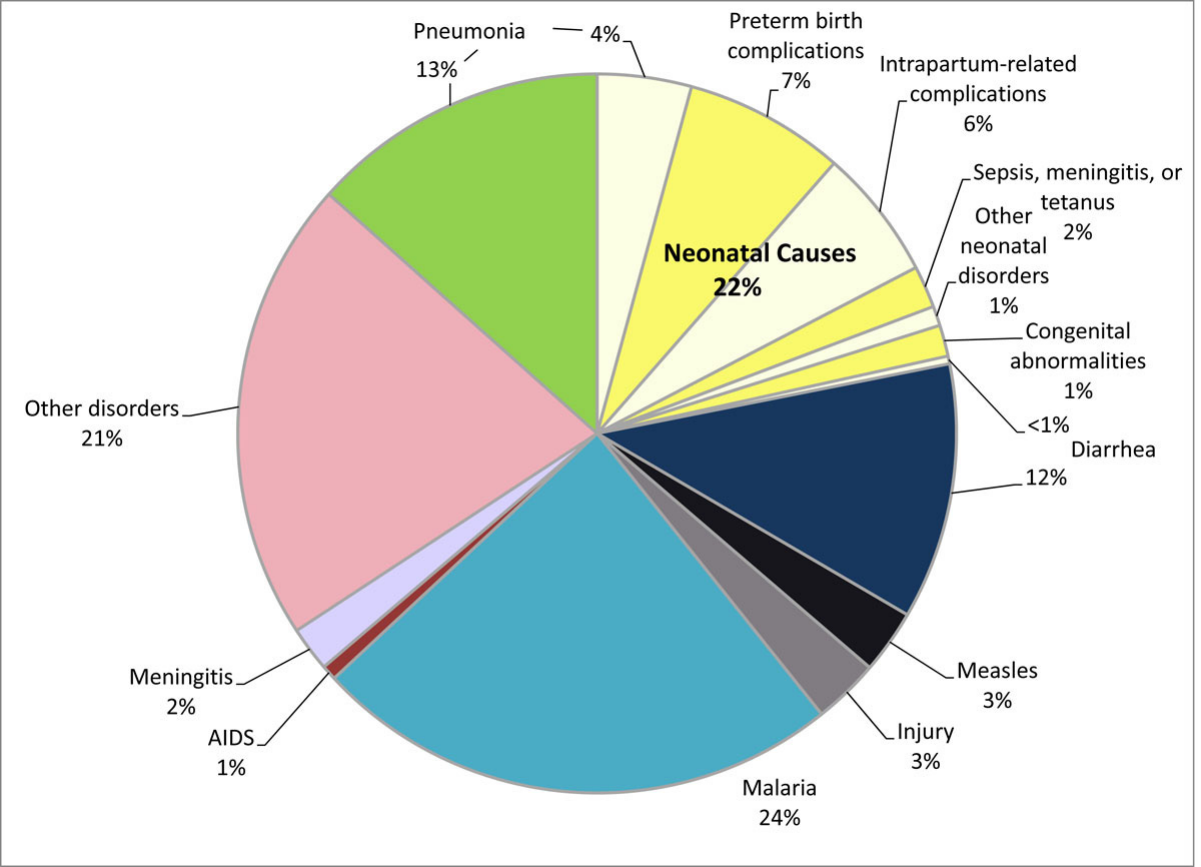
Causes of under-five mortality in Burkina Faso, 2010

Malaria, pneumonia, and diarrhea are important contributors to child deaths in Burkina Faso and globally, accounting for 24, 18, and 12 percent of under-five mortality in Burkina Faso and 7, 18, and 11 percent of under-five mortality globally, respectively [4]. Neonatal mortality accounts for 22 percent of deaths to children under five in Burkina Faso and 40 percent globally. Figure 1 shows the distribution of deaths in Burkina Faso by cause [4].

In 2008, the Ministry of Health (MoH) began a strategy to accelerate the scale-up of interventions with proven impact on maternal, neonatal, and child health. The program, called the Acceleration for Maternal, Neonatal and Child Health (“Acceleration”), is being implemented in nine districts in the North and Center North regions. Malaria treatment with artemisinin combination therapy (ACT) and diarrhea treatment with oral rehydration solution (ORS) and zinc are provided at the community level in all nine districts; treatment of pneumonia with oral antibiotics is provided in two districts as a pilot. By the end of 2010, all districts had trained volunteer community health workers to diagnose and treat malaria and diarrhea, and, in two districts, suspected pneumonia. In all but one district, where the drug kits were received in early 2011, community health workers had received drug kits. In addition to community case management (CCM) of childhood illness, the Acceleration targets the rapid scale-up of several other maternal, neonatal and child health interventions. Table 1 presents a list of all program interventions included in the Acceleration and their target coverage levels for 2013.

**Table 1:** Interventions for which coverage is being accelerated, including the indicator definition used in the model and the intervention target coverage

Coverage intervention	Indicator definition used in model	2013 Target
Antenatal Care	Proportion of live births in the previous 2 years for which the mother attended four or more antenatal care visits during the pregnancy (ANC4+).	80%
Pregnant women protected via intermittent preventive treatment of malaria (IPT) or sleeping under an insecticide-treated bed net (ITN)	Proportion of live births in the previous 2 years for which the mother received 2+ doses of SP/Fansidar during pregnancy.	70%
	Proportion of households that own at least one ITN.	
Skilled birth attendance (SBA)	Proportion of live births in the previous 2 years attended by a skilled attendant, including doctors, nurses, midwives or auxiliary midwives.	60%
Exclusive breastfeeding for the first six months of life	Proportion of children <1 month receiving only breast milk.	20%
	Proportion of children 1-5 months receiving only breast milk.	20%
Vitamin A Supplementation	Proportion of children 6-59 months of age receiving at least 1 dose of vitamin A during the last 6 months.	90%
Insecticide treated bed nets or indoor residual spraying	Proportion of households owning at least 1 insecticide treated bed net.	70%
Case management of diarrhea (ORS)	Proportion of children with suspected diarrhea treated with oral rehydration solution.	60%
Zinc for treatment of diarrhea	Proportion of children 6-59 months of age with suspected diarrhea treated with zinc	60%
Case management of pneumonia (oral antibiotics)	Proportion of children with suspected pneumonia treated with appropriate antibiotics.	50%
Case management of malaria (ACTs)	Proportion of children treated within 24 hours of the onset of fever in malaria endemic areas with an artemisinin combination therapy.	70%

An ongoing independent evaluation of the Acceleration seeks to assess whether under-five mortality is reduced by 25% at endline (2013) relative to baseline (2009) in the program areas, which is the program objective, and to what extent the reduction in mortality is attributable to the program. This paper uses the Lives Saved Tool (LiST) to model the impact of program scale-up upon under-five mortality rates using the measured baseline values and program targets. A previous analysis of the Acceleration using LiST was instrumental in establishing the intervention-specific targets for the program [5].

## Methods

### Lives Saved Tool (LiST)

LiST is a state-of-the-art modeling software package that uses available demographic and epidemiologic data to predict the effect that changes in coverage of health interventions will have on neonatal, under-five and maternal mortality [6]. It allows the user to model counterfactual scenarios to calculate not only the impact of a projected scale up of a health program, but also the impact relative to any number of alternate scenarios. LiST draws upon the technical expertise of the Child Health Epidemiology Reference Group (CHERG) [7] for estimation of key inputs, such as cause-specific mortality by country and intervention effectiveness. A description of the role of CHERG in the development of LiST is available elsewhere [8]. Supporting documentation for the interventions included in LiST is referenced within the software and available in a series of LiST journal supplements [8,9].

### Data sources

LiST requires baseline and end-line coverage levels of an intervention to project the effect of changes in coverage on under-five mortality. Intervention coverage measures the proportion of those needing an intervention who receive it. Examples of coverage indicators used in this analysis are shown in Table 1. Baseline coverage values were obtained from the LiST survey for the independent evaluation of the Acceleration conducted in 2010 [10]. The survey sampled 1,000 households in each of seven Acceleration districts and 2,000 households in each of the remaining two districts, Barsalogho and Gourcy, where CCM for pneumonia was implemented under the Acceleration. The survey also sampled 1,000 households in each of seven comparison districts with comparable demographic and health systems characteristics for a total of 18,000 households. The evaluation team hypothesized that the program would have a heightened impact in rural areas and therefore only sampled households from rural areas [10].

Baseline coverage values for LiST interventions came from the baseline survey report [10]. Where a LiST intervention was not included in the report, the coverage values were calculated by the authors from the survey database. See Annex Table 1 for a complete list of intervention coverage values from the baseline survey by district and region. Some LiST interventions (e.g., magnesium sulfate for pre-eclampsia, active management of the third stage of labor) are not readily measured through household surveys. In the absence of measured coverage data on these interventions, LiST estimates the coverage based on measured coverage of ANC4+ or of skilled birth attendance. We used this approach in our analysis as well.

Data before 2010 were needed to establish trends in coverage. The 2003 DHS [1] and 2006 Multiple Indicator Cluster Survey (MICS) [11] were used to establish prior coverage levels. The DHS sample included 9,097 households while the MICS sample included only 5,523 households. Both surveys were conducted nationally. The relatively larger sample size of the DHS allowed for the results to be stratified not only by urban or rural enumeration area but also by region. MICS data were also stratified by urban and rural area, but regional stratification was not possible. This analysis uses rural coverage values from both surveys to establish a trend leading up to 2010 for all projections. Regional data were not used for the reference period because the national rural coverage estimates from DHS 2003 and MICS 2006 were more representative of the Acceleration districts than regional estimates that include urban areas. Also, the lack of regionally-representative data in the MICS 2006 dataset would have further complicated the analysis, had regional data from the DHS been used.

Where available, coverage values from MICS were used preferentially to data from DHS as they were collected more recently. Where an estimated coverage value for the indicator of interest was only available in DHS, that value was used instead. An exception to this rule was made for the percentage of births attended by a skilled health worker. Both DHS and MICS included births attended by a trained traditional birth attendant (Matrone) when calculating this indicator, which is inconsistent with the current definition that excludes matrones [12]. The data in the MICS report could not be disaggregated to exclude matrones, so the indicator was recalculated from DHS as the sum of births attended by a doctor, nurse, or midwife.

### Analysis

Using data from DHS, MICS and the LiST survey, individual projections were created for each program district, region, and intervention group. A projection was also calculated for all 16 districts. Each projection began with the national DHS and MICS data to establish reference coverage values for either 2003 or 2006. The data from 2010 were then entered and interpolated with the values from 2006 or 2003.

In several instances this approach would have resulted in a modeled decrease in intervention coverage from the previous value through 2010. For these cases, we were concerned that the higher coverage value in 2003 or 2006 compared with 2010 might not reflect a real decrease in coverage over time. Instead, the higher value in 2003 or 2006 might have resulted from the use of national rural data from these surveys, rather than region- or district- level rural data. Where this conflict occurred, the coverage values from the LiST survey were considered more representative of the population of interest, and the 2010 values were used in place of the 2003 or 2006 survey values. Under this approach, for these interventions and districts we assumed there was no change in coverage in the projection prior to 2010, resulting in a flat secular trend for these interventions.

**Figure 2:**
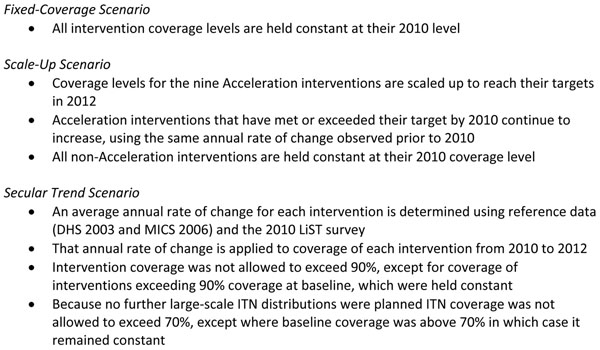
Summary of Fixed-Coverage, Scale Up, and Secular Trend Scenarios

Three scenarios were considered for the period 2010 to 2013 (Figure 2). First, the fixed-coverage scenario held coverage of all interventions constant at their 2010 level through 2013. Secondly, the scale-up scenario held coverage of all non-program interventions constant at their 2010 level and scaled up all program interventions to meet the MoH targets in 2013. In instances where the coverage target had been achieved by baseline, we assumed that coverage continued to increase to 2013, using the same annual rate of change observed prior to 2010. See Table 2 for baseline and endline coverage levels for program interventions used in the scale -up scenario by district. A final projection predicted the decrease in under-five mortality if the observed changes in intervention coverage during the reference period (2003 or 2006 through 2010) continued through 2013. These trends were calculated by applying the annual rate of change in coverage of an intervention prior to 2010 to the period 2010-2013. This projection assumed that once a secular trend caused coverage to reach 90%, then coverage of that intervention would cease to increase. Chloroquine for treatment of malaria was phased out of Burkina Faso beginning in 2005-2006, and use of ACTs was extremely limited at the time the 2006 MICS was conducted. The average annual rate of change for antibiotics for pneumonia was therefore used to project secular trends in treatment with ACTs from 2010 to 2013. All coverage changes were assumed to be linear.

**Table 2:** Percent reduction in under-five mortality from 2010 to 2013 with scale up of interventions, continuation of secular trends in coverage, and their difference

	Percent reduction in under-five mortality		
Projection	Scale Up	Secular Trends	Absolute difference*
**All Intervention and Comparison Districts**	22%	8%	14%

**Center North**	23%	6%	16%

Barsalogho	25%	9%	16%
Boulsa	19%	0%	19%
Kaya	20%	15%	6%
Kongoussi	22%	8%	14%
**North**	19%	6%	14%
Gourcy	20%	7%	13%
Ouahigouya	19%	16%	3%

Séguénéga	14%	11%	3%
Yako	17%	3%	14%
Titao	16%	3%	13%

**PMNCH without Pneumonia CCM**	18%	6%	11%

**PMNCH with Pneumonia CCM**	24%	9%	15%

*Absolute difference may not equal observed difference between columns due to rounding

This analysis created 42 unique projections: one fixed-coverage, scale-up, and secular trend projection for each district, region, intervention group, and all districts together. For each projection, LiST also estimated the change in under-five mortality from 2010 to 2013, and the change in mortality attributable to each intervention. Additional projections were created to examine the results of scaling up each intervention in isolation and scaling up all interventions simultaneously.

## Results

Table 3 shows the results of the Acceleration scale-up and secular trend scenarios, including the percent mortality reduction under these scenarios and the difference in mortality reduction between the two scenarios. The fixed-coverage scenario predicted minimal reductions in mortality related to vaccine herd effects in four districts. In all other districts there was no reduction in mortality (data not shown).

Scaling up all program interventions to their target levels by 2013 showed a 14-25% reduction in under-five mortality in the program districts. The reduction in mortality under the scale-up scenario exceeded the reduction in mortality predicted by the secular trend scenario (Table 2). Districts with lower baseline coverage of program interventions, especially the treatment interventions, were more likely to experience larger reductions in mortality under the scale-up scenario, as shown in Barsalogho and Kongoussi districts (25% and 22% reductions, respectively). Districts where baseline coverage of the treatment interventions was higher, such as Titao and Séguénéga, experienced relatively smaller reductions in mortality.

**Table 3:** Reduction in under-five mortality when all interventions are scaled up together

	Baseline coverage	Target Coverage	Change in Coverage	Percent Reduction in Under Five Mortality Due to Intervention
Antimalarials - artemisinin compounds for malaria	26%	70%	44%	10%
ORS - oral rehydration solution	23%	60%	37%	4%
Oral antibiotics : case management of pneumonia in children	33%	60%	27%	3%
Zinc – for treatment of diarrhea	4%	60%	56%	1%
Breastfeeding practices	33%	38%	5%	1%
ITN/IRS - Ownership of insecticide treated nets (ITN/LLIN) or household protected with indoor residual spraying	64%	70%	6%	<1%
Labor and delivery management^a^	71%	79%	8%	<1%
IPTp - Pregnant women protected via intermittent preventive treatment of malaria during pregnancy or by sleeping under an ITN	38%	70%	32%	<1%
Vitamin A supplementation	89%	90%	1%	<1%
Immediate assessment and stimulation^a^	56%	58%	2%	<1%
Clean birth practices^a^	60%	64%	4%	<1%
Syphilis detection and treatment^a^	23%	64%	41%	<1%
Total				22%

**Note:** Coverage of skilled birth attendance and exclusive breastfeeding during the first six months are not listed above because targets for these interventions had been achieved at baseline.

^a^ Increase in coverage of labor and delivery management, immediate assessment and stimulation, clean birth practices, and syphilis detection and treatment results from scaling up coverage of mothers receiving at least four antenatal care visits from 45% in 2010 to 80% in 2012.

The difference between the reduction in under-five mortality as a result of scaling up the program interventions and the reduction in under-five mortality due to secular trends in coverage change provides an approximation of the percentage reduction in mortality attributable to the program. There was large variation in program-attributable mortality reductions in mortality by district, with the largest reductions in Boulsa, Barsalogho, Kongoussi and Titao districts. Scaling up the program interventions in these districts accounted for reductions in under-five mortality that were 14 to 19 percentage points greater than the anticipated reductions in under-five mortality due to secular trends.

Table 3 shows the results for the intervention-specific reductions in mortality in all 16 districts if the program targets are met. The three interventions responsible for the largest reduction in mortality were case management of malaria, diarrhea, and pneumonia. The reductions in mortality that would be achieved by scaling up the interventions individually are very similar to the intervention-specific contributions to reduced mortality from scaling up all interventions simultaneously.

Each of the scale up projections was also examined to determine the intervention-specific contributions to the reduction in mortality. In all district level projections (results not shown), treatment of diarrhea with ORS and malaria with ACTs accounted for the majority of the reduction in mortality. Scaling up coverage of ITNs and Vitamin A supplementation had a small impact on mortality in some districts but none in others. Reasons for these findings are explored in the following section.

## Discussion

The results of the scale up scenario predict a reduction in mortality in each of the program districts, although the magnitude of the reduction varies greatly between districts. The Ouahigouya and Séguénéga districts of the North region show lower reductions in mortality than the other districts. The reduced program impact in these districts is consistent with the higher baseline coverage levels of the treatment interventions in these districts. Similarly, the lower baseline coverage level of the treatment interventions in Barsalogho, Boulsa and Yako districts explains the increased program impact in these districts.

Case management of malaria with ACTs, diarrhea with ORS and pneumonia with oral antibiotics were the program interventions with the most impact wherever they were implemented. This is due in part to the close relationship between these interventions and the cause of death distribution in Burkina Faso, where malaria, diarrhea, and pneumonia account for 49% of under-five mortality. Interventions targeting maternal and neonatal mortality, such as antenatal care and intermittent preventive treatment of malaria during pregnancy (IPTp), had less impact on under-five deaths. Neonatal mortality accounts for a smaller proportion of deaths in Burkina Faso than globally (22% compared to 40%) [13]. Few of the interventions targeted neonatal mortality directly, and the baseline coverage levels of ANC and skilled attendance at delivery were relatively high. Several other interventions appeared to have a relatively small effect on under-five mortality, due to high baseline coverage of these interventions. Baseline coverage of exclusive breastfeeding among children younger than six months and Vitamin A supplementation already exceeded the program target for coverage in many districts. Although only one district, Titao, exceed the target coverage for ITNs of 70%, all other districts had a coverage gap of 20% or less.

A sensitivity analysis examined the potential reduction in under-five mortality if all interventions were scaled up to 90% coverage with the exception of Vitamin A supplementation, for which target coverage was already 90%. This scenario found a much larger reduction in under-five mortality than if interventions were only scaled up to their original targets (36% compared to 22%).

### Limitations

In the context of the program evaluation, a major limitation of this analysis is that the baseline data were collected in 2010, while program activities began in 2009. The evaluation team was not invited to make a first visit to the country until 2009, and was able to conduct the baseline survey only once funding became available. CCM of diarrhea, malaria, and pneumonia, which is the aspect of the program that distinguishes the program area from other parts of the country, was effective only at the end of 2010. Program activities such as integrated management of childhood illness training and vitamin A campaigns had begun in 2009 prior to the survey; these activities were also ongoing nationwide. Therefore, this 2010-2013 analysis may not fully capture changes in the coverage of interventions such as vitamin A supplementation that are attributable to the program, and may underestimate the mortality impact.

These findings should be considered within the framework of LiST as a model that relies heavily upon user-defined assumptions for determining the trajectory of coverage scale up. The assumptions used were determined to be the most appropriate for the analysis, but the limitations of these assumptions should be explored to understand any potential bias that they might introduce into the results. The method used to determine the secular trend may be sufficient for drawing a simple comparison, but could be improved upon. The assumption of linearity for scale up associated with secular trends in coverage is unlikely to be true. Instead, scale-up curves are likely to be intervention-specific. For example, the scale up of long-lasting insecticide treated bed nets (LLINs) is likely to be a step function. A quantity of LLINs will be distributed in a campaign, as occurred in Burkina Faso in 2010, resulting in a rapid increase in coverage, but then coverage will increase much more slowly until the next LLIN campaign. Therefore increases in ITN coverage prior to 2010 will not necessarily continue at the same rate after 2010. This conflicts with the modeled secular trend for ITN coverage that showed large increases for most districts between 2010 and 2013.

These projections are also limited by the available coverage data before 2010. The 2003 Burkina Faso DHS and 2006 Burkina Faso MICS were representative of rural areas at national level and were not representative at district level like the results of the 2010 baseline survey. Additional data would be required to generate improved estimates of secular trends at district level, such as coverage estimates during the period leading up to 2010. Improved availability of data from this period would also allow for more accurate modeling of potential decreases in coverage prior to 2010, which were not considered in this analysis.

One critique of LiST is that the projected mortality rates are presented as point estimates with no confidence interval. The demographic and coverage data used to project population trends are estimated from survey data, and intervention effectiveness data are estimated from study results. Country-specific cause of death structures are also modeled estimates. An uncertainty analysis tool for LiST is in development and will be included in the software in the future.

## Conclusions

This analysis sought to assess the potential mortality reduction resulting from the Acceleration program in two regions of Burkina Faso. The results suggest that a reduction in under-five mortality ranging from 14% to 25% could be achieved by 2013, if program targets are met. The decrease in mortality over and above the modeled mortality reduction due to secular trends is somewhat smaller. Although these achievements will not meet the overall Acceleration goal for the nine intervention districts, they represent an important reduction in child deaths. In order to achieve the greatest reduction in under-five mortality, the MoH and its partners should continue their efforts to scale up these proven interventions to target levels of coverage and beyond.

## List of abbreviations used

ACT: Artemesisin combination therapy; CCM: Community case management; CHERG: Child Health Epidemiology Reference Group; DHS: Demographic and Health Survey; IPTp: Intermittent preventative treatment of malaria during pregnancy; LiST: Lives Saved Tool; MICS: Multiple Indicator Cluster Survey; MoH: Ministry of Health; ORS: Oral rehydration solution.

## Competing interests

The authors declare that they have no competing interests.

## Authors' contributions

AM and JB conceived of this study. AM did the analysis with advice from MM. AM developed the first draft which was then revised by MM, KG, and JB. All authors reviewed the final manuscript.

## Supplementary Material

Additional file 1 **Annex Table 1** Baseline coverage of the LiST indicators included in program scale up Click here for file

## Declaration

The publication costs for this supplement were funded by a grant from the Bill & Melinda Gates Foundation to the US Fund for UNICEF (grant 43386 to "Promote evidence-based decision making in designing maternal, neonatal, and child health interventions in low- and middle-income countries”). The Supplement Editor is the principal investigator and lead in the development of the Lives Saved Tool (LiST), supported by grant 43386. He declares that he has no competing interests.

This article has been published as part of *BMC Public Health* Volume 13 Supplement 3, 2013: The Lives Saved Tool in 2013: new capabilities and applications. The full contents of the supplement are available online at http://www.biomedcentral.com/bmcpublichealth/supplements/13/S3.
